# The Phase III CONNEX programme assessing the efficacy and safety of iclepertin in patients with schizophrenia: Trial design and recruitment update

**DOI:** 10.1192/j.eurpsy.2024.224

**Published:** 2024-08-27

**Authors:** C. Reuteman-Fowler, Z. Blahova, S. Ikezawa, S. Marder, P. Falkai, J. H. Krystal

**Affiliations:** ^1^Boehringer Ingelheim Pharmaceuticals Inc., Ridgefield, CT, United States; ^2^Boehringer Ingelheim RCV GmbH & Co. KG, Vienna, Austria; ^3^The University of Tokyo, Komaba, Meguro-ku, Tokyo, Japan; ^4^David Geffen School of Medicine, Los Angeles, CA, United States; ^5^Ludwig Maximilians University Munich, Munich, Germany, Munich, Germany; ^6^Yale University School of Medicine, New Haven, CT, United States

## Abstract

**Introduction:**

In a 12-week, Phase II (NCT02832037) trial, iclepertin (BI 425809), an inhibitor of glycine transporter-1, was generally well tolerated and significantly improved cognition in 509 patients with schizophrenia.

**Objectives:**

The Phase III CONNEX programme aims to confirm the efficacy, safety and tolerability of iclepertin in improving cognition and functioning across a larger cohort of patients with schizophrenia.

**Methods:**

The CONNEX programme includes 3 randomised, double-blind, placebo-controlled parallel group trials in patients with schizophrenia (NCT04846868, NCT04846881, NCT04860830) receiving stable antipsychotic treatment. Each trial aims to recruit ˜586 patients, 18–50 years old, treated with 1–2 antipsychotic medications (≥12 weeks on current drug and ≥35 days on current dose before treatment) who have functional impairment in day-to-day activities and interact ≥1 hour per week with a designated study partner. Patients with cognitive impairment due to developmental, neurological or other disorders, with a current DSM-5 diagnosis other than schizophrenia or receiving cognitive remediation therapy within 12 weeks prior to screening, will be excluded. Patients will be recruited from multiple centres across 41 countries in Asia, North and South America, Europe and the Asia-Pacific Region, and randomised 1:1 to receive either iclepertin 10 mg (oral administration; n=293), or placebo (n=293) once daily for 26 weeks. The primary endpoint is change from baseline in overall composite T-score of the Measurement and Treatment Research to Improve Cognition in Schizophrenia Consensus Cognitive Battery. The key secondary endpoints are change from baseline in total score on the Schizophrenia Cognition Rating Scale and change from baseline in the adjusted total time T-score in the Virtual Reality Functional Capacity Assessment Tool.

**Results:**

The CONNEX programme is currently recruiting (**Table**); the first patients were enrolled in Aug–Sept 2021 and completion is expected in Q1 2025. The presentation will describe the current study status, information relating to screening failures, and the experience of collecting these data as part of a large multi-country, multicentre study.

**Table.** The number of patients recruited by 31 August 2023

	CONNEX 1	CONNEX 2	CONNEX 3
**Screened**	565	521	493
**Randomised**	409	360	350
**Completed trial medication**	202	184	191

**Conclusions:**

Iclepertin may represent the first efficacious medication for cognitive impairment associated with schizophrenia.

**Funding:**

Boehringer Ingelheim

**Disclosure of Interest:**

C. Reuteman-Fowler Employee of: Boehringer Ingelheim, Z. Blahova Employee of: Boehringer Ingelheim, S. Ikezawa Consultant of: Boehringer Ingelheim Pharma GmbH, Lundbeck, Takeda Pharma, Sumitomo Dainippon Pharma, Employee of: International University of Health and Welfare, Mita Hospital, Tokyo, Japan, S. Marder Consultant of: Boehringer Ingelheim Pharma GmbH, Merck, Biogen and Sunovion, P. Falkai Consultant of: Boehringer Ingelheim Pharma GmbH, Boehringer Ingelheim Pharma Advisory Board, J. H. Krystal Shareolder of: Freedom Biosciences, Inc., Biohaven Pharmaceuticals, Sage Pharmaceuticals, Spring Care, Biohaven Pharmaceuticals Medical Sciences, EpiVario, RBNC Therapeutics, Terran Biosciences and Tempero Bio, Consultant of: Aptinyx, Atai Life Sciences, AstraZeneca Pharmaceuticals, Biogen, Biomedisyn Corporation, Bionomics, Boehringer Ingelheim International, Cadent Therapeutics, Clexio Bioscience, COMPASS Pathways, Concert Pharmaceuticals, Epiodyne, EpiVario, Greenwich Biosciences, Heptares Therapeutics, Janssen, Jazz Pharmaceuticals, Otsuka America Pharmaceutical, Perception Neuroscience Holdings, Spring Care, Sunovion Pharmaceuticals, Takeda Industries, Taisho Pharmaceutical Co.; Biohaven Pharmaceuticals, BioXcel Therapeutics, Cadent Therapeutics, Cerevel Therapeutics, Delix Therapeutics, EpiVario, Eisai, Jazz Pharmaceuticals, Novartis, PsychoGenics, RBNC Therapeutics, Tempero Bio and Terran Biosciences Advisory Boards

